# Recurrent Hypoglycaemia in a Patient with Metastatic Pancreatic Carcinoma

**DOI:** 10.1371/journal.pmed.0030331

**Published:** 2006-08-29

**Authors:** Ronald C. W Ma, Raymond S. K Lo, Morris H. L Tai, Juliana C. N Chan, Chun Chung Chow, Jean L. F Woo

**Affiliations:** Case report from **1** Department of Medicine and Therapeutics, Prince of Wales Hospital, Chinese University of Hong Kong, Shatin, Hong Kong, Special Administrative Region, People's Republic of China; 2 Department of Medicine and Geriatrics, Shatin Hospital, Shatin, Hong Kong, Special Administrative Region, People's Republic of China; 3 Department of Chemical Pathology, Prince of Wales Hospital, Chinese University of Hong Kong, Shatin, Hong Kong, Special Administrative Region, People's Republic of China

## Abstract

The patient's recurrent hypoglycaemia was found to be due to non-islet cell tumour hypoglycaemia.

## Presentation of Case

A 79-y-old man presented with obstructive jaundice in July 2001 and was diagnosed with carcinoma of the head of the pancreas. He underwent a total pancreatectomy, as well as resection of the distal stomach, duodenum, spleen, and gall bladder. Histology showed a 9 × 10 × 6.5–cm tumour at the head of the pancreas that, upon microscopic examination, showed a moderately differentiated invasive adenocarcinoma displaying a mixed papillary and glandular growth pattern. The resection margins were free of tumour, and all resected lymph nodes were clear of metastases. The patient developed diabetes following his pancreatectomy and was started on insulin. His diabetes was managed with human isophane insulin injections, 18 units in the morning, 6 units in the evening with HbA1c of 8.2% (normal range 5.1%–6.4%).

**Figure 1:**
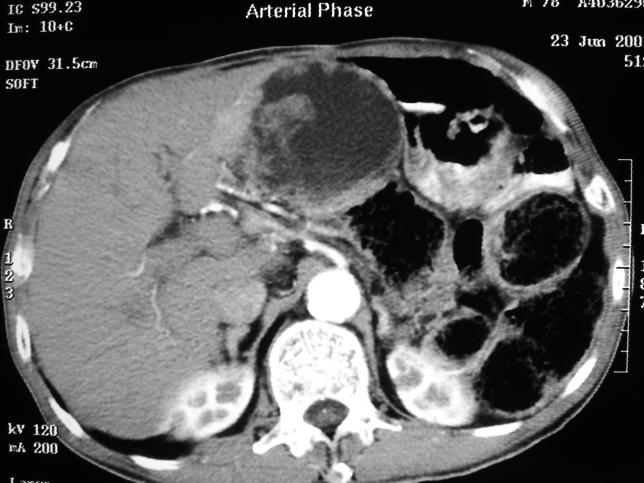
Enhanced Computed Tomography Scan of Abdomen Enhanced computed tomography scan of abdomen showing heterogenous liver metastases with irregular rim enhancement as well as hypodense lesions in the portal region suggestive of metastatic lymphadenopathy.

In June 2003, the patient was noted to have hepatomegaly on follow-up and was found to have liver and portal lymph node metastases on a computed tomography scan of the abdomen (Figure 1). The result of the carbohydrate antigen 19–9 test was elevated at 139 U/ml (normal <37 U/ml). Metastatic adenocarcinoma was diagnosed, and the patient opted for conservative management.

In January 2004, the patient was admitted following an episode of loss of consciousness. He was diagnosed with hypoglycaemia with an unrecordable haemastix reading on arrival and a random plasma glucose level of 2.9 mmol/l after receiving intravenous dextrose. His renal function tests were normal, and liver function tests were normal except for an elevated alkaline phosphatase of 116 IU/l (normal 40–100 IU/l). A short synacthen test was normal. During admission, the patient had further episodes of documented hypoglycaemia with plasma glucose down to 1.8 mmol/l. His insulin dose was therefore reduced to isophane insulin 12 U once daily.

The patient was admitted again in March 2004, following an episode of confusion during which he stabbed himself with a knife. He underwent emergency surgery for haemostasis and removal of the knife. A psychiatric evaluation after his operation found no evidence of depression or suicidal ideation.

The patient was transferred to the palliative care ward and was noted to have recurrent episodes of hypoglycaemia despite progressive reduction of his insulin dose to isophane insulin 4 U daily. He was on no other medications except frusemide for mild ankle oedema. Liver function tests were normal except for a mildly elevated alkaline phosphatase of 144 IU/l (normal 40–100 IU/l). The international normalised ratio was 1.26 (normal range 0.9–1.1). His chest x-ray was normal.

In view of the recurrent episodes of symptomatic hypoglycaemia, he was eventually taken off all insulin injections. A fasting blood sample taken 2 d after stopping insulin showed blood glucose 5.4 mmol/l, C-peptide <0.5 μg/l (normal range 1.1–5 μg/l), and insulin level <2 mIU/l (normal range 6–35 mIU/l). Repeated urine analysis was negative for ketones.

10 d after stopping insulin, the patient developed further episodes of hypoglycaemia, with blood glucose levels of 1.7 mmol/l and 2.1 mmol/l. Insulin levels were undetectable. A repeat short synacthen test showed a raised baseline cortisol of 1,082 nmol/l (reference range 171–536 nmol/l) and post-stimulation cortisol of 1,145 nmol/l, indicating activation of the hypothalamic-pituitary-adrenal axis from the recurrent hypoglycaemia. Insulin-like growth factor (IGF)-I level was <2 nmol/l (normal range 5–22.5 nmol/l). Total IGF-II was 35.6 nmol/l. The IGF-II/IGF-I ratio was raised, at >18 (normal <10). The IGF-BP3 level was low, at 0.4 mg/l (normal 0.7–4.4 mg/l). Growth hormone (GH) level was undetectable.

The patient was diagnosed as having non-islet cell tumour hypoglycaemia (NICTH) due to metastatic adenocarcinoma. His hypoglycaemic episodes were stabilized with an intravenous dextrose infusion, and the patient remained relatively free of neuropsychiatric symptoms or psychospiritual distress. The patient eventually died of cancer cachexia 2 wk later. An autopsy was not performed.

## Discussion

Spontaneous hypoglycaemia is suggested by the presence of Whipple's triad: symptoms suggestive of hypoglycaemia, biochemical confirmation of hypoglycaemia, and reversal of symptoms following correction of hypoglycaemia. Neuroglycopenic symptoms tend to predominate as autonomic warning symptoms, and counterregulatory hormonal responses are often impaired in the setting of recurrent hypoglycaemia [1].

Hypoglycaemia in patients with cancer may be secondary to medications, liver failure, renal failure, severe sepsis, or adrenal insufficiency. In addition, NICTH is increasingly recognized as an important cause of hypoglycaemia in such patients. NICTH has been reported to occur in association with a variety of malignant tumours, including hepatomas, colorectal carcinomas, breast carcinomas, renal cell carcinomas, and lung carcinomas, as well as haematological malignancies and benign tumours such as mesenchymal fibromas [2].

The occurrence of NICTH in association with adenocarcinoma of the pancreas is extremely rare [3], but dramatically illustrates the hypoglycaemic potential of the condition. In our patient, who had undergone a total pancreatectomy resulting in insulin deficiency and diabetes, the tumour was able to cause reversal of his diabetes and induce recurrent hypoglycaemia, even in the absence of any endogenous insulin secretion.

**Figure 2:**
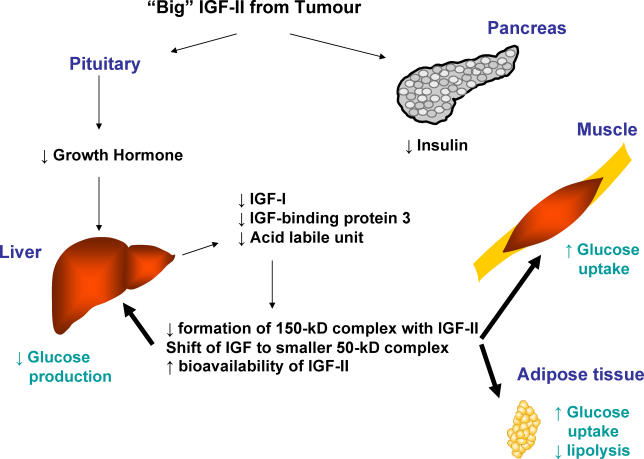
Pathophysiology of NICTH

The hypoglycaemia in NICTH is believed to be due to tumour production of a prohormone form of IGF-II, termed “big” IGF-II. IGF-II is normally sequestered and inactivated by binding to specific binding proteins such as IGF-BP3 to form a 150-kD ternary complex. The elevated IGF-II levels inhibit the secretion of endogenous insulin and GH. The low GH levels in NICTH in turn result in decreased levels of IGF-BP3, thereby impairing the formation of the ternary complexes (Figure 2). As a result, a larger proportion of IGF-II can circulate freely as binary complexes, which causes hypoglycaemia by inhibiting hepatic glucose production and enhancing glucose disposal to muscle [2].

The diagnosis of NICTH can be confirmed by finding suppressed insulin, C-peptide, and GH concentration in the setting of hypoglycaemia (confirmed by laboratory measurement of blood glucose), along with elevated total and “big” IGF-II levels. A total IGF-II/IGF-I ratio of >10 is thought to be diagnostic. It is important to take blood for the above measurements (and in some cases, to screen for sulphonylurea) at the time of symptomatic hypoglycaemia, and to document symptoms and the response to glucose treatment. Adrenal insufficiency due to adrenal metastases should be excluded.

Surgical resection is the treatment of choice for tumours causing hypoglycaemia due to NICTH, and should result in normalization of blood glucose and the biochemical abnormality. However, this may not be possible in patients with metastatic disease. Debulking the tumour with radiotherapy or chemotherapy has been reported to be effective in controlling the hypoglycaemia [4]. If surgery is not feasible, the hypoglycaemia can be treated with repeated glucagon injections [5] or intravenous glucose. Glucocorticoids are very effective in preventing hypoglycaemia and have been shown to reduce tumour production of “big” IGF-II in a dose-dependent manner [6]. A dose of prednisolone 30–60 mg/d or dexamethasone 4 mg/d has been reported to be effective [7]. Recent advances in understanding the pathophysiology of NICTH should be helpful for clinicians in the management of recurrent hypoglycaemia in patients with cancer.

## Supporting Information

Alternative Language Text S1 Chinese Translation of the Full Text by RCWM (136 KB PDF)

## Abbreviations

GH: growth hormone
IGF: insulin-like growth factor
NICTH: non-islet cell tumour hypoglycaemia

## Notes

Learning Points Hypoglycaemia in patients with known malignancy may be due to medications, liver failure, renal failure, sepsis, adrenal insufficiency, or NICTH. In the investigation of spontaneous hypoglycaemia, it is important to document hypoglycaemia with a reliable laboratory blood glucose measurement and to take blood at the time of hypoglycaemia for measurement of insulin, C-peptide, and other hormones before treatment with glucose has been given. NICTH can occur in association with a variety of malignant tumours, but can also occur secondary to haematological malignancies and some benign mesenchymal tumours. The hypoglycaemia that occurs in NICTH is not dependent on endogenous insulin secretion, but instead is caused by tumour production of a prohormone form of IGF-II. Unprovoked hypoglycaemia or unexplained changes in insulin sensitivity in a patient with known malignancy should alert physicians to the possibility of NICTH.
