# Supplementary material for: Recurrent Hypoglycaemia in a Patient with Metastatic Pancreatic Carcinoma
Source: PLoS Med. 2006 Aug 29;3(8):e331. doi: 10.1371/journal.pmed.0030331 (PMC1564285; doi:10.1371/journal.pmed.0030331)
Supplement: Alternative Language Text S1 — (136 KB PDF) [file pmed.0030331.sd001.pdf]

轉移性胰臟腺癌及低血糖症

馬青雲 [1, 2*]

盧思傑 [2]

戴學良 [3]

陳重娥 [1]

周振中 [1]

胡令芳 [1, 2]

來自 **1** 香港中文大學威爾斯親王醫院內科及藥物治療學系、**2** 香港沙田醫院內科及老人科、**3** 香港中文大學威爾斯親王醫院化學病理學系的病例報告

* 來信請註明通信地址。電子郵件：rcwma@cuhk.edu.hk

病例描述

一名七十九歲男性患者於 2001 年 7 月出現阻塞性黃疸，並被診斷為胰頭癌。該患者進行過全胰臟切除術以及胃遠端、十二指腸、脾臟和膽囊切除手術。組織學檢查顯示胰頭部位元有一 9 × 10 × 6.5 釐米大小的腫瘤，經顯微鏡檢查顯示為中度分化的浸潤性腺癌，呈乳頭狀和腺樣增生。切除部位的邊緣無腫瘤，所有切除的淋巴結都沒有轉移灶。該患者在胰臟切除術後出現了糖尿病，並開始注射胰島素治療糖尿病，早上給予 18 個單位、晚上給予 6 個單位的低精蛋白人胰島素注射劑，HbA1c 的平均值為 8.2%（正常範圍為 5.1%–6.4%）。

2003 年 6 月，隨訪時該患者被診斷患有肝腫大，並在腹部 CT（電腦斷層攝影術）掃描時發現該患者具有肝和肝門淋巴結轉移灶（圖 1）。癌抗原（CA）19-9 試驗結果表明 CA 19-9 的水平升高至 139 U/ml（正常值<37 U/ml）。該患者被診斷為轉移性腺癌，並選擇保守治療。

2004 年 1 月，該患者突然失去意識被送入院接受治療。入院時，該患者被診斷為低血糖，血糖讀數低於血糖計的下限。在接受靜脈注射葡萄糖治療後，隨機

葡萄糖血漿濃度仍只有 2.9 mmol/l。腎功能測試正常，肝功能測試除鹼性磷酸酶 116 IU/l（正常範圍為 40–100 IU/l）有所升高外，其餘均正常。短時間促皮質素測試（short synacthen test）正常。入院期間，患者進一步出現有記錄的低血糖徵狀，血糖降至 1.8 mmol/l。因此，患者的胰島素劑量被下調至每日一次 12 個單位的低精蛋白胰島素。

該患者於 2004 年 3 月出現意識混亂後再次入院，在意識混亂期間該患者使用小刀刺傷了自己。該患者進行了緊急手術以便止血，並取出了小刀。手術後精神科醫生對他進行的精神評估並沒有發現任何抑鬱或自殺意念的證據。

該患者及後被轉移至安寧護理病房。儘管其胰島素劑量被逐漸降至每天 4 個單位的低精蛋白胰島素，但是他仍反復出現低血糖徵狀。除給予呋塞米治療輕度踝水腫之外，他沒有服用其他藥物。肝功能測試除鹼性磷酸酶 144 IU/l（正常範圍為 40–100 IU/l）有所升高外，其餘均正常。國際標準化比值 (INR) 為 1.26（正常範圍 0.9–1.1）。該患者的胸部 X 光檢查正常。

鑒於該患者症狀性低血糖徵狀反復發作，最後停用了所有的胰島素注射劑。停用胰島素 2 天後採集的禁食血液樣品顯示血糖濃度 5.4 mmol/l、C 肽濃度<0.5 µg/l（正常範圍 1.1–5 µg/l）以及胰島素濃度<2 mIU/l（正常範圍 6–35 mIU/l）。重複尿液分析顯示甲酮呈陰性。

停用胰島素後 10 天，該患者進一步出現低血糖徵狀，血糖水平處於 1.7~2.1 mmol/l 之間。胰島素水平極低。重複的短時間促皮質激素試驗顯示考的松基線濃度為 1,082 nmol/l（參考範圍 171–536 nmol/l），刺激後考的松的濃度為 1,145 nmol/l，相對正常平日水平有所增高，這表明反復低血糖導致丘腦-垂體-腎上腺軸的啟動。胰島素類生長因數（IGF）-I 的水平<2 nmol/l（正常範圍 5–22.5 nmol/l）。IGF-II 總體濃度為 35.6 nmol/l，IGF-II/IGF-I 的比率有所提高，大於 18（正常範圍<10）。IGF-BP3 水平較低，為 0.4 mg/l（正常範圍 0.7–4.4 mg/l）。生長激素（GH）的水平也是很低。

該患者被診斷為因轉移性腺癌而引起的非胰島細胞腫瘤低血糖（NICTH）。在給予靜脈葡萄糖注射劑穩定低血糖發作之後，患者再無心理精神憂慮症狀。2 周後，該患者最後死於癌症引起的併發症。沒有進行屍解。

討論

惠普耳氏三聯征（Whipple’s triad）的三項病徵表明患者患有自發性低血糖：這包括出現低血糖的症狀和徵兆、血糖數值低下以及給糾正低血糖後症狀立即緩解。低血糖性神經症狀往往成為主要病徵，因為在低血糖反復發作的情況下，自動警惕症狀及逆調節激素反應（counterregulatory hormonal responses）通常會被減弱 (1)。

癌症患者的低血糖可能是繼發於藥物、肝功能衰竭、腎衰竭、嚴重敗血症或腎功能不全的反應。除此以外，越來越多的學者陸續認同 NICTH 是該類患者體內低血糖的重要原因。NICTH 與多種惡性腫瘤有關，其中包括肝癌、結腸直腸癌、乳房癌、腎細胞癌、肺癌以及血液系統惡性腫瘤和良性腫瘤如間質細胞纖維瘤 (2)。

胰腺癌導致 NICTH 是極罕有的(3)，但這個病例清楚地反映了 NICTH 能夠降低血糖的力量。上述的病人，儘管接受過全胰臟切除術而導致胰島素缺乏和糖尿病，即使在沒有內源性胰島素分泌的情況下，腫瘤也能夠逆轉糖尿病，而且更導致復發性低血糖。

學者現在認為 NICTH 引發的低血糖是由於腫瘤分泌跟 IGF-II 相似的激素原型（稱為大分子量 IGF-II）而引起的。通常情況下，IGF-II 是通過與特殊結合蛋白如 IGF-BP3 結合形成 150-kD 三價複合物而被螯合且被滅活。IGF-II 水平的升高會抑制內源性胰島素和 GH 的分泌。之後，NICTH 引起的低 GH 水平會導致 IGF-BP3 的水平降低，因此會影響三價複合物的生成（圖 2）。因此，較多的 IGF-II 就可以作為二價複合物自由迴圈，這就會通過抑制肝臟內的葡萄糖生成和提高肌肉的葡萄糖消耗而引發低血糖(2)。

可在（通過實驗室血糖測定進行確診）低血糖情況下通過檢測受抑制的胰島素、C 肽和 GH 的濃度以及升高的總體和大分子 IGF-II 濃度來確診 NICTH。IGF-II/IGF-I 總比率大於 10 即被認定具有診斷意義。最重要的是，一旦出現症狀性低血

糖時，應該盡被抽取血液樣本進行如上的測定，並記錄進行低血糖治療的症狀和反應。在某些病例，可能還要檢查有沒有進食過磺尿素。

手術切除是治療誘發 NICTH 型低血糖的腫瘤的最有效療法，同時可以使血糖和生物化學異常指標恢復正常。但是，患有轉移性疾病的患者可能無法進行手術切除。有報告稱用放射療法或化學療法進行的減瘤手術在控制低血糖方面也可有效(4)。如果手術治療不可行，那麼可反復給予升血糖素注射劑(5)或靜脈注射葡萄糖治療低血糖。皮質激素在預防低血糖方面非常有效，且已有實驗表明皮質激素能夠依劑量有效地降低腫瘤分泌大分子 IGF-II (6)。報告指潑尼松龍 30–60 mg/d 的劑量或地塞米松 4 mg/d 的劑量較有效(7)。能夠瞭解 NICTH 病理生理學的最新進展，對於臨床醫生治療癌症患者反復性低血糖應該很有幫助。

重點

- 患有已知惡性腫瘤的患者若出現低血糖徵狀可能是由藥物、肝功能衰竭、腎衰竭、敗血症、腎功能不全或 NICTH 引發的。

▪ 在自發性低血糖的評估中，必須在給予葡萄糖進行治療之前使用可靠的實驗室血糖測試方法記錄低血糖，並在出現低血糖徵狀時採集血液樣本以測定胰島素、C 肽及其它激素。

▪ NICTH 與多種惡性腫瘤有關，但亦可能是繼發於血液系統惡性腫瘤以及一些良性間質細胞癌而產生的。

▪ 出現在 NICTH 內的低血糖不依賴於內源性胰島素的分泌，相反，而是由腫瘤分泌 IGF-II 激素原型生成而引起的。

▪ 若患有已證實惡性腫瘤的患者無故出現低血糖或者胰島素敏感性出現無法解釋的變化，那麼醫生應該警惕 NICTH 的可能性。

參考文獻

1. Mitrakou A, Fanelli C, Veneman T, Perriello G, Calderone S, et al. (1993) Reversibility of unawareness of hypoglycemia in patients with insulinomas. N Engl J Med 329: 834–839.
2. Zapf J, Futo E, Peter M, Froesch ER (1992) Can “big” insulin-like growth factor II in serum of tumor patients account for the development of extrapancreatic tumor hypoglycemia? J Clin Invest 90: 2574–2584.

3. Sturrock ND, Selby C, Hosking DJ (1997) Spontaneous hypoglycaemia in a noninsulin-dependent diabetes mellitus patient with disseminated pancreatic carcinoma. Diabet Med 14: 324–326.
4. Kishi K, Sonomura T, Sato M (1997) Radiotherapy for hypoglycaemia associated with large leiomyosarcomas. Br J Radiol 70: 306–308.
5. Hoff AO, Vassilopoulou-Sellin R (1998) The role of glucagon administration in the diagnosis and treatment of patients with tumor hypoglycemia. Cancer 82: 1585–1592.
6. Baxter RC, Holman SR, Corbould A, Stranks S, Ho PJ, et al. (1995) Regulation of the insulin-like growth factors and their binding proteins by glucocorticoid and growth hormone in nonislet cell tumor hypoglycemia. J Clin Endocrinol Metab 80: 2700–2708.
7. Teale JD, Wark G (2004) The effectiveness of different treatment options for non-islet cell tumour hypoglycaemia. Clin Endocrinol (Oxf) 60: 457–460.

1. **Mitrakou A, Fanelli C, Veneman T, et al.** 1993 Reversibility of unawareness of hypoglycemia in patients with insulinomas. N Engl J Med 329:834-9
2. **Zapf J, Futo E, Peter M, Froesch ER** 1992 Can "big" insulin-like growth factor II in serum of tumor patients account for the development of extrapancreatic tumor hypoglycemia? J Clin Invest 90:2574-84
3. **Sturrock ND, Selby C, Hosking DJ** 1997 Spontaneous hypoglycaemia in a noninsulin-dependent diabetes mellitus patient with disseminated pancreatic carcinoma. Diabet Med 14:324-6
4. **Kishi K, Sonomura T, Sato M** 1997 Radiotherapy for hypoglycaemia associated with large leiomyosarcomas. Br J Radiol 70:306-8

5. **Hoff AO, Vassilopoulou-Sellin R** 1998 The role of glucagon administration in the diagnosis and treatment of patients with tumor hypoglycemia. Cancer 82:1585-92
6. **Baxter RC, Holman SR, Corbould A, Stranks S, Ho PJ, Braund W** 1995 Regulation of the insulin-like growth factors and their binding proteins by glucocorticoid and growth hormone in nonislet cell tumor hypoglycemia. J Clin Endocrinol Metab 80:2700-8
7. **Teale JD, Wark G** 2004 The effectiveness of different treatment options for non-islet cell tumour hypoglycaemia. Clin Endocrinol (Oxf) 60:457-60
